# Digital phenotyping, behavioral sensing, or personal sensing: names and transparency in the digital age

**DOI:** 10.1038/s41746-020-0251-5

**Published:** 2020-03-25

**Authors:** David C. Mohr, Katie Shilton, Matthew Hotopf

**Affiliations:** 1 Center for Behavioral Intervention Technologies, Department of Preventive Medicine, Northwestern University, Chicago, IL USA; 2 College of Information Studies, University of Maryland, College Park, College Park, MD USA; 3 King’s College London, Institute of Psychiatry, Psychology and Neuroscience, London, UK

**Keywords:** Ethics, Medical research

## Abstract

Data from networked sensors, such as those in our phones, are increasingly being explored and used to identify behaviors related to health and mental health. While computer scientists have referred to this field as context sensing, personal sensing, or mobile sensing, medicine has more recently adopted the term digital phenotyping. This paper discusses the implications of these labels in light of privacy concerns, arguing language that is transparent and meaningful to the people whose data we are acquiring.

Common networked devices like the smartphone (e.g., GPS, keyboard touches, phone use, and communication patterns) and wearables can provide a continuous stream of the data about an individual’s behaviors, psychological states, and environments, forming a picture of their lived experience^1^. This sensing technology can, with varying degrees of accuracy, estimate sleep patterns, activity, and social engagement, as well as mental health conditions^2^. The application of sensing technology has enormous potential to improve our understanding of the experience of individuals and our capacity to deliver behavioral health treatments. Behavioral markers inferred from sensed data are beginning to be integrated into apps, making them simpler and more engaging to use^3,4^. Such sensing apps can be integrated into standard psychological or behavioral treatments^5^, or delivered as stand-alone or coached interventions^6^. Passive tracking of populations of at-risk people could facilitate early identification and intervention for behavioral problems. These potential clinical innovations have led to a rapidly growing field of research, and are beginning to be developed commercially, thereby supporting their dissemination.

As with any emerging field, there have been many different terms used to describe this application of sensing technology. The exploration of the use of phone sensors to estimate behaviors, psychological states, and environmental contexts began more than 15 years ago in computer science, where it has been referred to variously as context sensing, reality mining, mobile sensing, behavioral sensing, and personal sensing^2^. As medicine entered the field, the name “digital phenotyping” was proposed in 2015^7^, and has rapidly gained currency, becoming the most commonly used term in publications listed in PubMed. The term digital phenotyping has been adopted by funders, including the US National Institute of Health and the Wellcome Trust. From the research world, the term is spreading into publications for the healthcare industry, as well as into general media such as the New York Times^8^, and is now used by companies that are commercializing these technologies. As sensing technology for health and mental health becomes disseminated through commercialization and general media, it is incumbent upon us to consider the implications of the labels we use to describe it.

The language in a name provides information to an audience, thereby framing how that audience understands the product or service. The term digital phenotyping speaks to a medical audience, whose oldest texts, written in Greek, provide terms still used today, such as dyspnea (bad breathing) and melancholia (originally black bile). The term digital phenotyping (to show a type) provides a good description for a medical audience of the aims and processes involved in using digital traces to identify characteristics of an individual. It helps contextualize the field of sensing within medicine, which provides legitimacy, and suggests how to integrate sensing into genetics, diagnosis, and prognosis^9^.

But what might the term digital phenotyping signal mean to those whose data are being used? That such sensing is medical and scientific, perhaps? That it is complex? It does not convey to the average person that we are engaging in a sensitive form of surveillance: collecting large amounts of data, and using those data to understand deeply personal things, such as how they sleep, where they go, how and when they communicate with others, or whether they may be experiencing a mental health condition.

Yet, these are the people to whom we most need to explain the risks of participation, and why they should trust us with their data. These data are incredibly revealing, and we are asking research participants and commercial users to be vulnerable to our decision-making. For example, in a study of GPS data from the phones of 1.5 million Europeans, it took only four GPS points over 15 h to identify 95% of individuals^10^. As we detect behaviors and mental health conditions using sensed data, which often include GPS, the behaviors and conditions we detect can be linked directly to individuals, even without traditional personal identifiers. This capability is emerging in an environment where some companies in the digital health industry have demonstrated a remarkable lack of regard for privacy. A recent study, which intercepted the network traffic generated in the use of the top 30 mental health and smoking cessation apps, found that more than 80% of the apps shared data for advertising and marketing purposes, but only 28% disclosed this in a privacy policy^11^. Thus, the field of sensing poses significant vulnerabilities in a context that has tended to exploit rather than protect the people we aim to help.

To earn participant trust, the labels we use should increase, not decease, the transparency of our intent (what we are doing and why) and practice (how we are getting the data and its nature)^12^. Among the terms used in computer science, mobile sensing, behavioral sensing, and personal sensing come closest to providing this information. “Sensing” conveys an automated, background data collection. “Mobile” suggests the device (as in mobile phone), and that the data gathered are not restricted to a single place. “Behavioral” identifies the target of sensing. “Personal” conveys the intimate nature of the behaviors, and states that we are attempting to detect.

Terminology in modern medicine has trended toward transparency^13^. Standard English has often been used in naming more recent innovations. “Bypass surgery” is descriptive and understandable (even if the modifier “coronary” is less so). “Scanning” gives people a general sense of the aims and processes for imaging technologies. The use of a descriptor, such as personal sensing for emerging data-gathering technologies, would be well aligned with the growing use of descriptive English terms in medicine.

Sensing technology targeting health and mental health is making its way into our lives and healthcare systems. It has enormous potential to enhance behavioral healthcare, but it is not without risks. The use of language that is transparent about the intent and practice behind this technology with the people whose data we are using is both ethically responsible and more likely to engender trust over time. For this emerging technology, our colleagues in computer science had it right when they selected terms using standard English descriptors that provide average people with an understanding of the intent and practice. We urge the field to use terms that are easily understood by the people whose data we are using. Our preference has been for the term “personal sensing.” It conveys the intent and practice, as well as the personal nature of the behaviors and states we are attempting to detect. By using standard English, we demonstrate respect for the people we are trying to serve and support transparency of our practices, which has not been uniformly provided in digital health and mental health.
